# ENGAGING CHINESE AND KOREAN AMERICAN COMMUNITIES IN DEMENTIA RESEARCH: A JOURNEY OF INCLUSIVITY AND PARTNERSHIP

**DOI:** 10.1093/geroni/igae098.0378

**Published:** 2024-12-31

**Authors:** Jing Wang, Mary Mittelman, I Tek Leong, Sung Ji Park, Xiang Qi, Yaolin Pei, Cynthia Epstein, Bei Wu

**Affiliations:** University of New Hampshire, Durham, New Hampshire, United States; New York University, New York, New York, United States; New York University, New York, New York, United States; New York University, New York, New York, United States; New York University, New York, New York, United States; University of Texas at Austin, Texas, United States; New York University, New York, New York, United States; New York University, New York, New York, United States

## Abstract

The NYUCI-ES Project, designed to deliver a family-based psychosocial intervention to enhance social support and reduce cardiometabolic disease risk for Chinese and Korean American dementia caregivers, has developed and undertaken a culturally sensitive recruitment strategy. Our navigation through the journey has shed light on the challenges and successes faced when recruiting in these minority and underrepresented communities. Common challenges include reluctance of involving other family members, stigma towards dementia and the concept of family counseling. Encountered challenges prompted us towards multiple efforts to engage the communities, including leveraging online communities, building strategic partnerships with local community organizations, participate in local events and make presentations at local service agencies, and working with community partners to adapt recruitment messages and methods to better align with cultural norms and preferences. For example, we replaced the term “counseling” with “family meetings” and used “memory issues” rather than “dementia” and emphasized the practical benefits and the opportunity for children to practice filial piety. For the Chinese community, our strategies focused on initiating the conversations with practical skills rather than directly discussing emotions. For the Korean community, framing the intervention in terms of discussing caregiving strategies while highlighting the personal and social benefits of participating in the study has helped lower the rejection rate. Our journey highlights the importance of culturally sensitive recruitment strategies in dementia research and demonstrates the potential for greater engagement and participation when communities are approached with respect, sensitivity, and an understanding of their unique cultural dynamics.

